# Device-free isolation of photoreceptor cells from patient iPSC-derived retinal organoids

**DOI:** 10.1172/jci.insight.186338

**Published:** 2025-06-12

**Authors:** Nicholas E. Stone, Laura R. Bohrer, Nathaniel K. Mullin, Alexander Berthold, Allison T. Wright, Ian C. Han, Edwin M. Stone, Robert F. Mullins, Budd A. Tucker

**Affiliations:** 1 Institute for Vision Research,; 2 Department of Ophthalmology and Visual Sciences, and; 3 Department of Neuroscience and Pharmacology, Carver College of Medicine University of Iowa, Iowa City, Iowa, USA.

**Keywords:** Ophthalmology, Stem cells, Translation, iPS cells

## Abstract

Autologous photoreceptor cell replacement is one of the most promising strategies currently being developed for the treatment of patients with inherited retinal degenerative blindness. Induced pluripotent stem cell–derived (iPSC-derived) retinal organoids, which faithfully recapitulate the structure of the neural retina, are an ideal source of transplantable photoreceptors required for these therapies. However, retinal organoids contain other retinal cell types, including bipolar, horizontal, and amacrine cells, which are unneeded and may reduce the potency of the final therapeutic product. Therefore, approaches for isolating fate-committed photoreceptor cells from dissociated retinal organoids are desirable. In this work, we present partial dissociation, a technique that leverages the high level of organization found in retinal organoids to enable selective enrichment of photoreceptor cells without the use of specialized equipment or reagents such as antibody labels. We demonstrate up to 90% photoreceptor cell purity by simply selecting cell fractions liberated from retinal organoids during enzymatic digestion in the absence of mechanical dissociation. Since the presented approach relies on the use of standard plasticware and commercially available current good manufacturing practice–compliant reagents, we believe that it is ideal for use in the preparation of clinical photoreceptor cell replacement therapies.

## Abstract 2

This paper describes an inexpensive, clinically translatable photoreceptor isolation strategy for the production of cell therapies to treat inherited blindness.

## Introduction

In several forms of inherited retinal degeneration, gradual loss of photoreceptor cells in a patient’s retina leads to progressive vision loss. While gene therapy shows promise for halting disease progression if a molecular diagnosis is available, restoration of vision will likely require photoreceptor cell replacement (1–8). One promising source of transplantable photoreceptors is induced pluripotent stem cell–derived (iPSC-derived) retinal organoids. We and others have developed 3D retinal differentiation protocols, which produce retinal organoids that recapitulate the structure of the human retina and contain fate-committed photoreceptor cells (9–19). Our group is particularly interested in using patient-derived iPSC lines to produce autologous photoreceptor cell therapies, which we believe will eliminate the need for long-term immune suppression following transplantation. We have produced current good manufacturing practice (cGMP) protocols for both the generation and differentiation of patient-derived iPSCs and are exploring methods by which production can be scaled to clinically relevant levels in an academic environment (9, 10, 20).

While clinical-grade production of patient-derived retinal organoids containing transplantable photoreceptor precursor cells is now possible, to enhance potency of the therapeutic product, a method for isolating photoreceptor cells is required. Specifically, transplantation of inner retinal neurons, which typically remain in patients with inherited retinal degeneration, is not likely to contribute to the desired clinical outcome and may interfere with photoreceptor cell integration with host bipolar cells. FACS and magnetic activated cell sorting (MACS) constitute gold standard cell-sorting technologies. Both are mature, are high throughput, and exhibit high selectivity, provided antibody labels can be targeted to unique cell surface antigens (or a combination of markers in the case of FACS) present on cells selected for enrichment or depletion (21–25). However, using animal-derived antibodies to target fluorescent probes or magnetic beads is not an ideal approach when isolating cells destined for human transplantation. Furthermore, processing multiple patient samples on single pieces of large, expensive equipment increases the risk of cross contamination as well as the cost of the resulting therapy. Thus, other GMP-compliant methods for enriching photoreceptor from nonphotoreceptor cells are highly desirable.

Retinal organoids faithfully reproduce the architecture of the mature human retina and exhibit a characteristic laminar structure in which the photoreceptors are found in the outermost layer. In this work, we evaluated the degree to which partial enzymatic digestion can be used to exploit this spatial segregation to enrich transplantable photoreceptor cells from patient iPSC-derived retinal organoids. Such an approach would decrease the regulatory burden of the resulting cell therapy by removing the need for xenobiotic sorting reagents, reduce cost by avoiding the use of specialized equipment, and increase the potency of the resulting cell therapy by enriching the cells that contribute to the desired clinical outcome.

## Results

As we have shown previously, substantial variability in terms of photoreceptor production capacity exists between patient-derived iPSC lines. To address this, over the course of this study, we have used 5 iPSC lines derived from 4 different patients. Information about these lines, including their genotypes and sex, are available in Supplemental Table 1 (supplemental material available online with this article; https://doi.org/10.1172/jci.insight.186338DS1).

**Figure 1:**
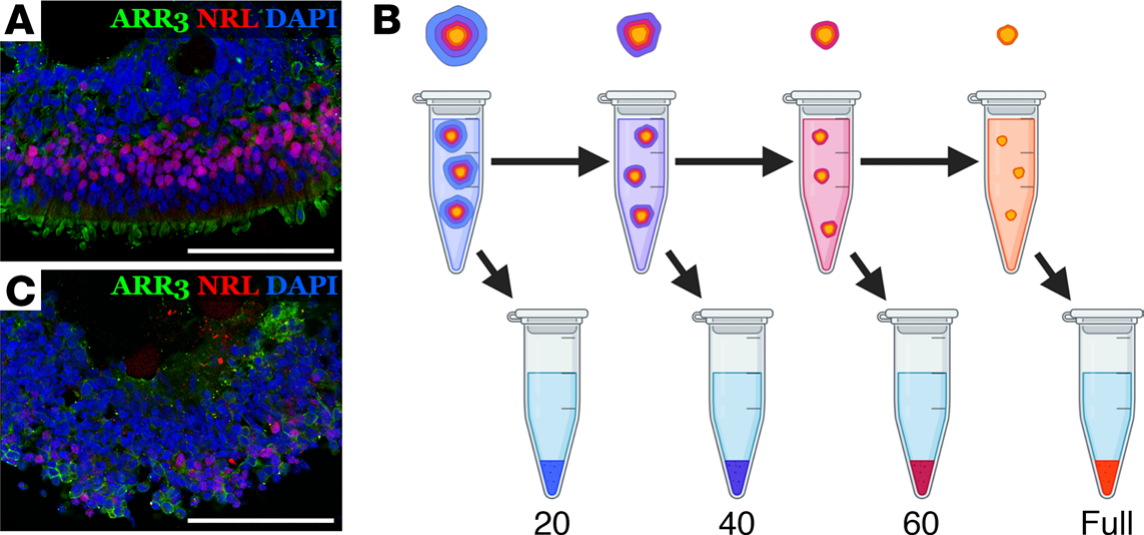
Overview. (**A**) Patient iPSC-derived retinal organoids (day 160) showing characteristic laminar structure with photoreceptors expressing NRL and ARR3 present on their outer layer. (**B**) Partial dissociations were performed by placing organoids in papain on a rotating rack at 37°C and periodically collecting liberated cells. For the scRNA-Seq experiments shown in Figures 4 and 5, fractions were collected every 20 minutes. Between each stage of dissociation, the supernatant containing liberated cells was removed, after which the organoids were rinsed and placed in fresh papain. After collecting the final partially dissociated cell fraction, remaining aggregates were fully dissociated via trituration. (**C**) After performing 40 minutes of dissociation, the outer layer of photoreceptors is selectively removed. Scale bars: 100 μm.

As shown in Figure 1A, retinal organoids produced using modern 3D differentiation protocols exhibit a characteristic laminar structure closely recapitulating the normal architecture of the neural retina. Our hypothesis was that, during enzymatic dissociation, the outer layer of photoreceptor cells would be liberated first, allowing for isolation of purified photoreceptor cells. To test this hypothesis, retinal organoids were incubated in papain, and liberated cells were collected at various time points for analysis. At each time point, retinal organoids were allowed to settle and the supernatant containing liberated cells was collected (Figure 1B). Remaining organoids were rinsed and placed in fresh papain for continued dissociation. After collecting the final partial dissociation fraction, the remaining solid organoids were mechanically dissociated via trituration with a 1 mL pipette tip. To illustrate our approach, in Figure 1C, we show selective removal of photoreceptors from mature line 1 retinal organoids after 40 minutes of dissociation.

**Figure 2:**
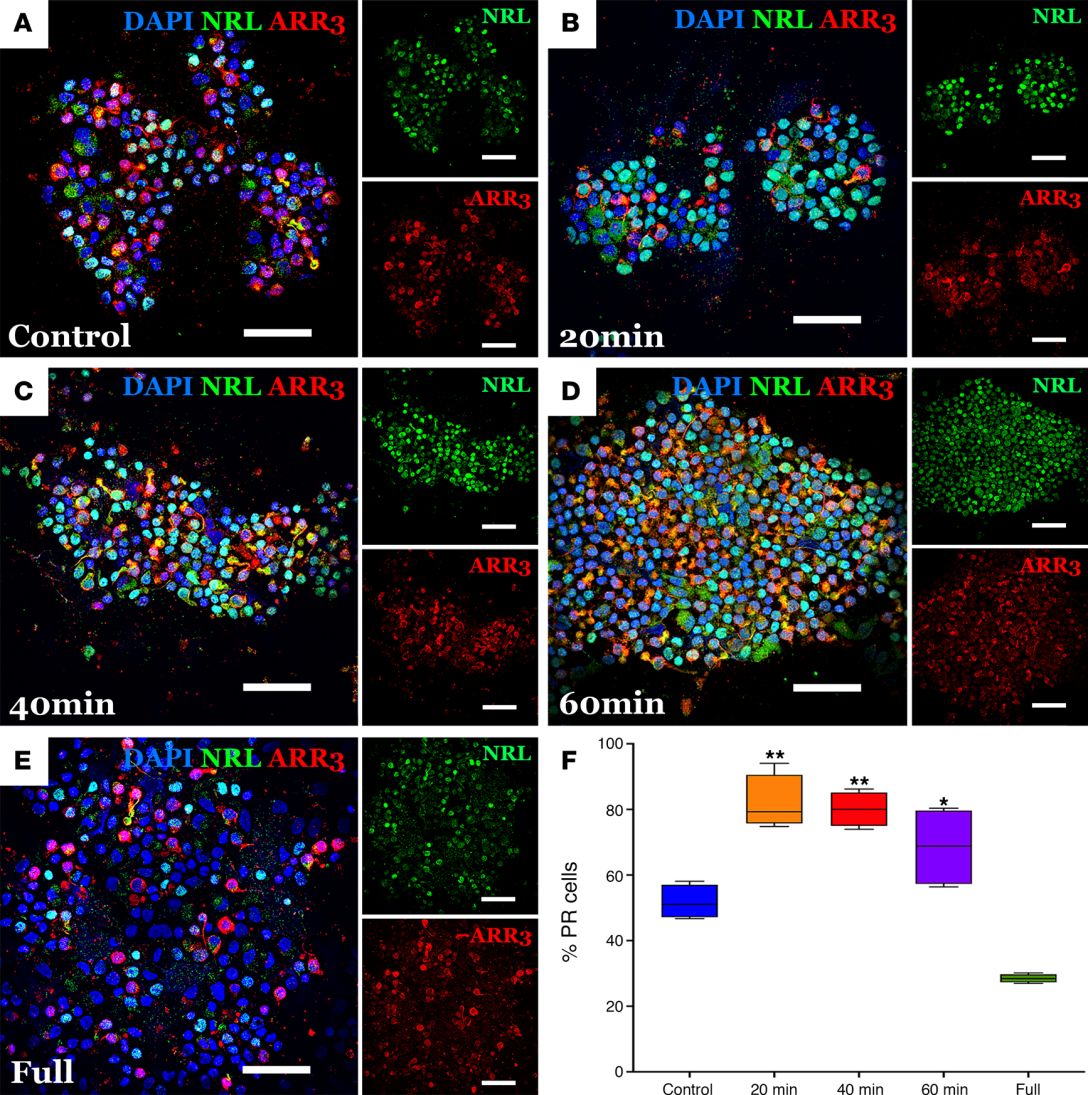
ICC of cells liberated from organoids via partial dissociation. Cells liberated from line 1 organoids were spun onto slides and stained for NRL and ARR3. (**A**–**D**) Cells were collected from a control one-step (**A**), complete dissociation as well as after 20 minutes (**B**), 40 minutes (**C**), and 60 minutes (**D**) of partial dissociation. (**E**) After 60 minutes of dissociation, the remaining solid aggregates were fully dissociated and stained. (**F**) Photoreceptor (PR) cell purity was assessed based on NRL and ARR3 staining. Raw counts of NRL^+^ and ARR3^+^ cells obtained by a skilled reader are available in Supplemental Table 3. Statistical significance was determined using a 1-way ANOVA with Tukey’s testing for post hoc comparisons. *P < 0.05, **P < 0.01.

To investigate whether partial dissociation can generate cell fractions enriched for intact, mature photoreceptors, we performed partial dissociations on line 1 organoids and used a CytoSpin instrument to centrifuge at 254*g* the cell fractions onto slides and assessed via immunocytochemistry (ICC). As shown in Figure 2, partial dissociation liberates cell populations that are enriched for neural retina-specific leucine zipper protein–positive (NRL^+^) rods and retinal cone arrestin-3–positive (ARR3^+^) cone photoreceptors as compared with a control dissociation. Furthermore, the cell suspension obtained by fully dissociating the remaining tissue was depleted for photoreceptors as compared with the control, suggesting the photoreceptor enrichment observed in the partially dissociated cells was not due to a difference in composition between the 2 groups of organoids. In addition, the presence of ARR3^+^ outer segment–like projections on the isolated cones shows that partial dissociation is a gentle procedure that causes minimal disruption of photoreceptor cells.

**Figure 3:**
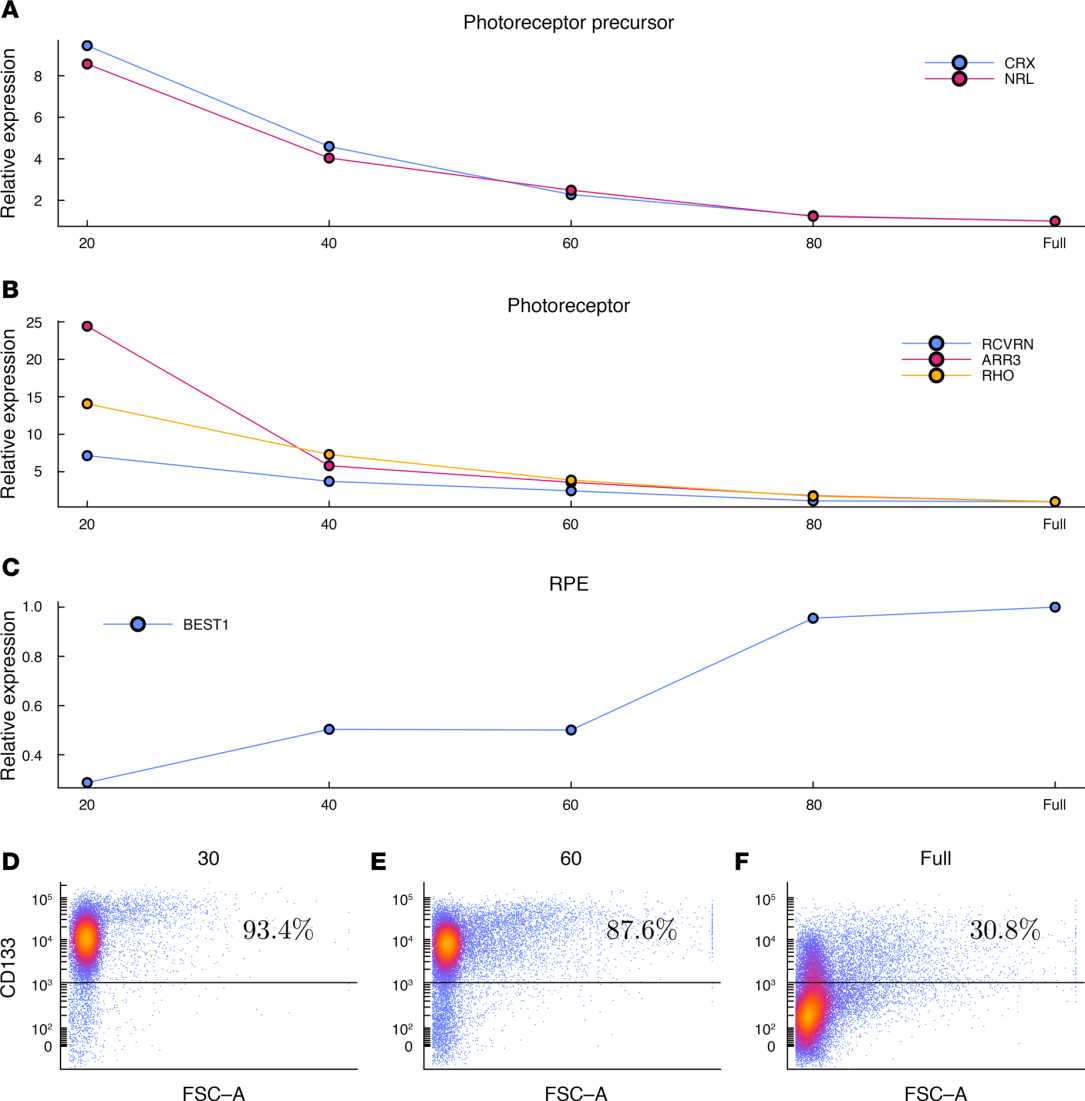
RNA and protein analysis of cells liberated during partial dissociation. (**A**–**C**) Expression of photoreceptor precursor (**A**), photoreceptor (**B**), and RPE markers (**C**) as assessed via qPCR in cell fractions obtained after 20, 40, 60, and 80 minutes of partial dissociation of line 2 organoids, as well as in the fully dissociated remaining tissue. (**D**–**F**) Relative abundance of CD133^+^ photoreceptors was also assessed via flow cytometry after partial dissociation of line 3 organoids for 30 minutes (**D**) and 60 minutes (**E**) as well as in fully dissociated remaining tissue (**F**). Raw CT and calculated relative expression values are provided in Supplemental Table 4.

In Figure 3, we present further characterization of cell samples obtained from partially dissociated retinal organoids via quantitative PCR (qPCR) and flow cytometry. The relative expression of genes associated with photoreceptors and committed photoreceptor precursors decreased with dissociation time in line 2 retinal organoids (Figure 3, A and B), and there was a corresponding increase in the expression of a retinal pigmented epithelium (RPE) marker (Figure 3C). Also, there was decreasing photoreceptor cell purity over dissociation time, as quantified via flow cytometry of partially dissociated line 3 organoids (Figure 3, D–F). Taken together, these results confirm what was shown in Figure 1: that during partial dissociation, the outer layer of retinal organoids are liberated first, allowing production of cell fractions enriched for photoreceptor cells.

**Figure 4:**
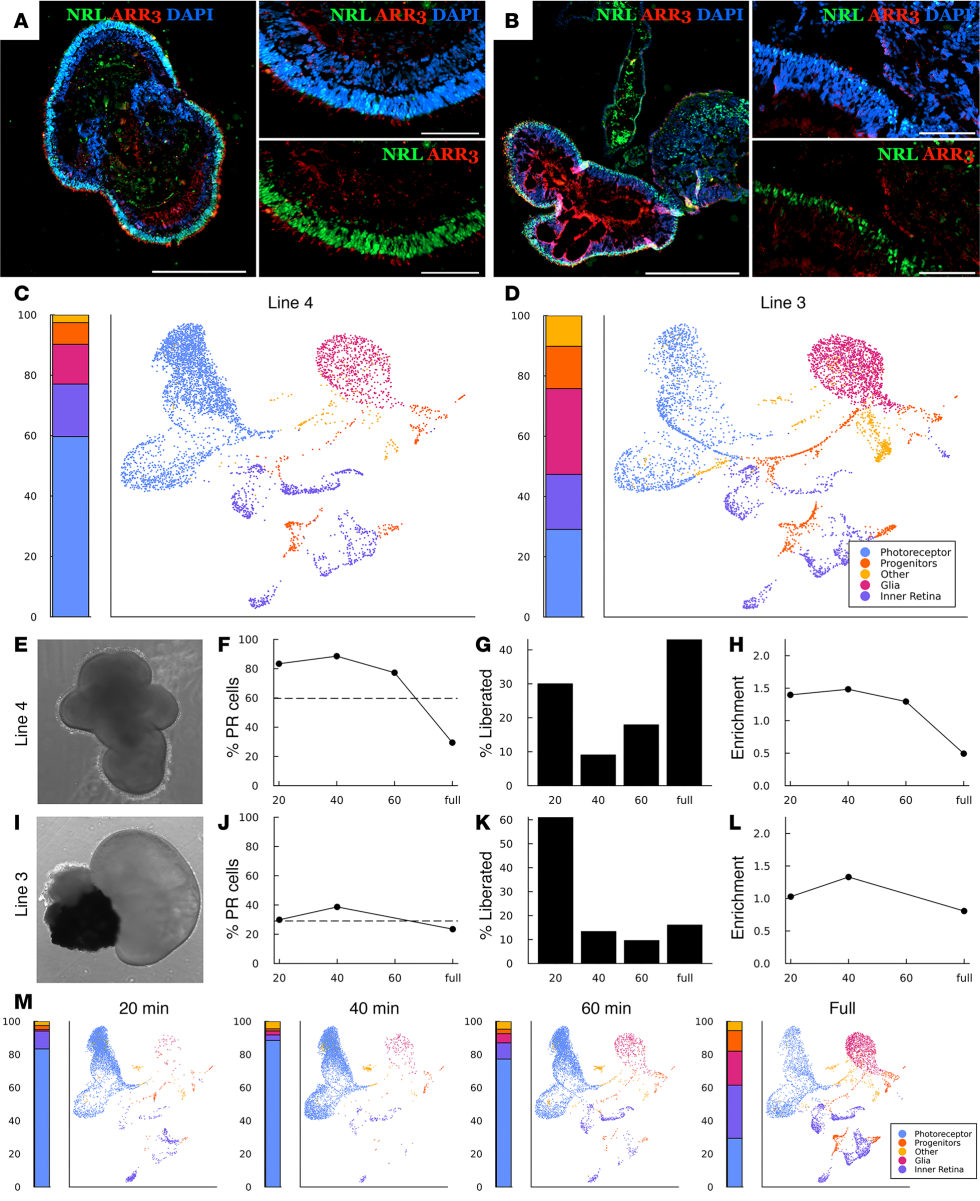
Partial dissociation of 2 patient lines. (**A** and **B**) Characteristic structure of well-organized line 4 organoids (**A**) and poorly organized line 3 organoids (**B**). Scale bars: 500 μm (primary panel), 100 μm (insets). (**C** and **D**) Bar and UMAP plots showing the relative proportion and gene expression patterns of cells collected during control dissociations of each cell line. (**E**–**L**) Photoreceptor purity and dissociation rate as a function of dissociation time. Each row shows a representative organoid (**E** and **I**) along with the photoreceptor purity (**F** and **J**) (dashed line shows the photoreceptor purity of a control dissociation of the same cell line), cell recovery (**G** and **K**), and photoreceptor enrichment (**H** and **L**) (fold change relative to a control dissociation) for each fraction recovered during staged partial dissociation. Cell fractions were taken after 20, 40, and 60 minutes of total dissociation time, after which the remaining aggregates were fully dissociated via trituration (full) and collected. (**M**) UMAP plots for line 4 demonstrating proportion of each cell type present in each dissociation fraction. The 60-minute time point for the purity and enrichment plots of **J** and **L** are missing due to the library preparation failing for that sample. UMAP plots for all samples and time points are available in Supplemental Figure 2. Relative proportions of annotated cell types are available in Supplemental Table 5. Raw cell counts used to prepare recovery plots are available in Supplemental Table 6.

For autologous photoreceptor cell replacement, iPSC-derived retinal organoids will need to be generated on a per-patient basis. While recent work has demonstrated the feasibility of this approach, variability between patient-derived iPSC lines can make clinical manufacturing difficult. Specifically, not all patient-derived iPSC lines are able to generate retinal organoids of the same quality or number (26). To address whether these patient-to-patient differences in retinal organoid quality have an effect on our partial dissociation photoreceptor enrichment strategy, as well as to further characterize the purity and identity of liberated cells, we performed partial dissociation on 2 lines and assessed the liberated cell fractions using single-cell RNA-Seq (scRNA-Seq). Line 4 is an efficient producer of laminated retinal organoids that contain many photoreceptor cells within their outermost layer (Figure 4, A and E). Line 3, however, while still an efficient retinal organoid producer, generally gives rise to organoids that are less well organized (i.e., lack of clear lamination) and often contain large aggregates of RPE (Figure 4, B and I). Despite these structural differences, both lines contained the same collection of cell types with similar gene expression patterns (Figure 4, C and D). After subjecting both lines to staged partial dissociation, we found that the well-organized line 4 yielded more cells in enriched fractions (i.e., fractions with photoreceptor cell purity greater than control dissociations) than the line 3 organoids (Figure 4, E–L). Importantly, relatively large numbers of cells were recovered in these enriched fractions, indicating that this enrichment strategy is capable of isolating photoreceptor cells at reasonable total yield, leaving nonphotoreceptor inner retinal cells behind (Figure 4M). While a substantial number of cells was recovered from the enriched fractions of both lines, there is a difference between their dissociation kinetics. In the well-organized line, many photoreceptor cells were liberated in the first fraction, after which the dissociation rate slowed before eventually increasing again (Figure 4G). One possible explanation for this behavior is that dissociation stalled when the outer plexiform layer (OPL) beneath the outer layer of photoreceptor cells was reached. We hypothesize that the less-organized line 3 does not exhibit the same dissociation kinetics because the attached clusters of RPE and general disorganization affects the integrity of the OPL (e.g., once the RPE cluster is removed, the inner layers of the organoid are exposed, allowing nonphotoreceptor cells to escape).

**Figure 5:**
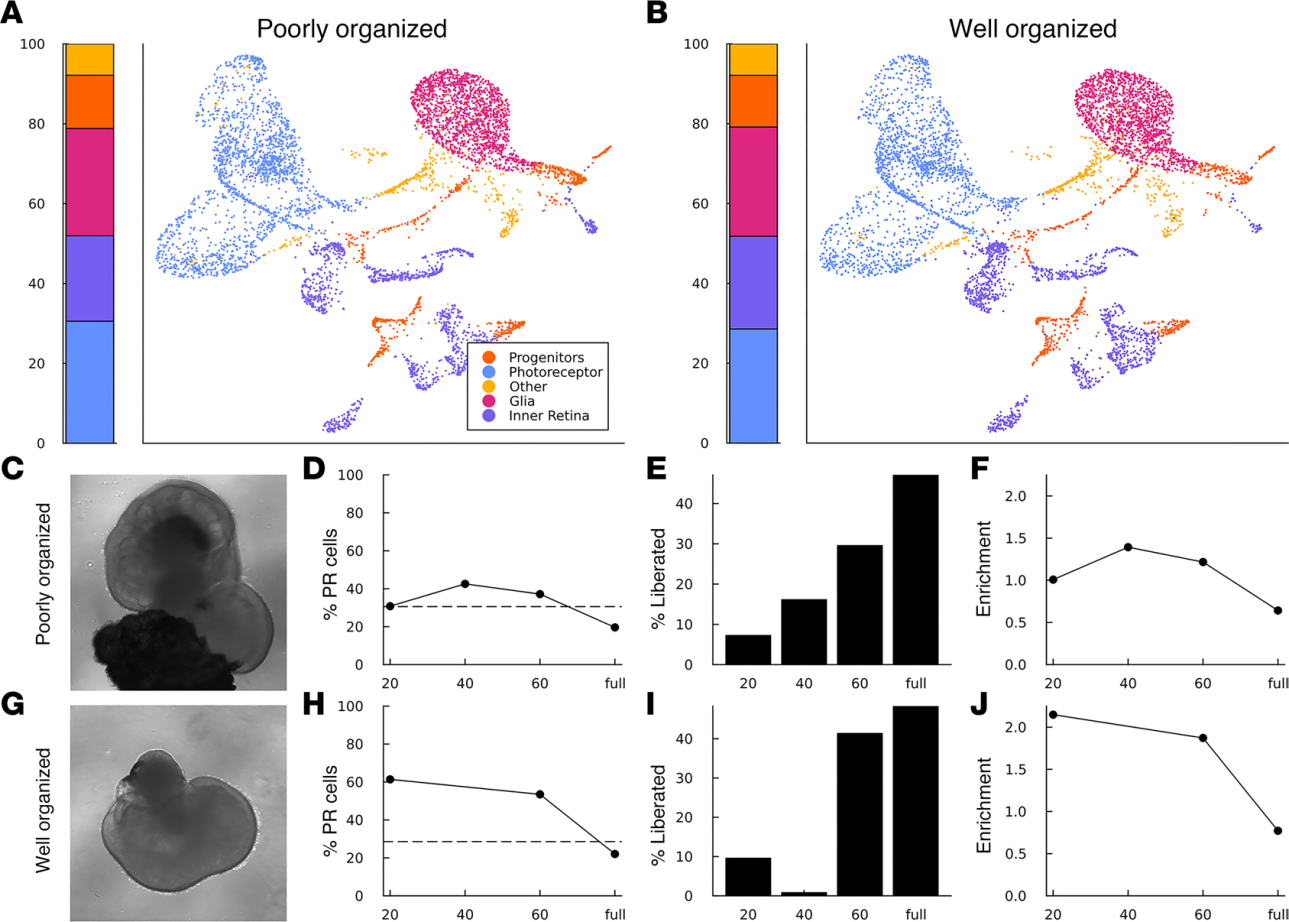
Partial dissociation of a patient line presorted by organoid organization. Prior to performing staged partial dissociation, line 5 organoids produced during a single round of differentiation were sorted into “well-organized” and “poorly organized” groups. (**A** and **B**) Bar and UMAP plots showing the relative proportion and gene expression patterns of cells collected during control dissociations of each group of organoids. (**C**–**J**) Photoreceptor purity and dissociation rate as a function of dissociation time. Each row shows a representative organoid (**C** and **G**) along with the photoreceptor purity (**D** and **H**) (dashed line shows the photoreceptor purity of a control dissociation of the same cell line), cell recovery (**E** and **I**), and photoreceptor enrichment (**F** and **J**) (fold change relative to a control dissociation) for each fraction recovered during staged partial dissociation. Cell fractions were taken after 20, 40, and 60 minutes of total dissociation time, after which the remaining aggregates were fully dissociated via trituration (full) and collected. The 40-minute time point is missing from the purity and enrichment panels (**H** and **J**) due to not enough cells being collected for scRNA-Seq analysis. UMAP plots for all samples and time points are available in Supplemental Figure 2. Relative proportions of annotated cell types are available in Supplemental Table 5. Raw cell counts used to prepare recovery plots are available in Supplemental Table 6. Representative ICC of day 160 line 5 organoids is available in Supplemental Figure 3.

To confirm that the differences in maximum purity and enrichment that we observed between line 3 and line 4 were due to organoid structure, we proceeded to perform partial dissociations on organoids generated from line 5, which produces both well-organized (clearly laminated with a distinct outer nuclear layer, ONL) and poorly organized (lack of clearly distinct ONL and large clusters of RPE) retinal organoids. While both the poorly organized and well-organized populations contained the same relative proportions of the constituent cell types, the well-organized organoids yielded dissociation fractions that were more highly enriched for photoreceptor cells (Figure 5). Furthermore, the well-organized sample showed the same characteristic dissociation kinetics as line 4 (Figure 5I) whereas the poorly organized sample did not (Figure 5E), indicating that this signature is likely a result of the highly organized structure of the organoids as hypothesized above. While less efficient in terms of total yield, these results also indicate that our partial dissociation approach could be used to produce highly enriched photoreceptor cell fractions from patient lines that produce organoids with varying levels of organization by manually presorting organoids prior to dissociation.

## Discussion

Photoreceptor cell replacement therapies show great promise for restoring vision in patients with many forms of currently untreatable retinal degenerative blindness. cGMP-compliant 3D differentiation protocols that can produce retinal organoids from patient-derived iPSCs have been developed (9–19). In these 3D differentiation approaches, the development of retinal organoids mimics that of the human retina in utero, resulting in laminated tissue containing committed photoreceptor lineages, which are desired for transplant, along with other retinal cell types. Given that only photoreceptors are expected to contribute to the therapeutic outcome of photoreceptor replacement therapies, techniques capable of enriching photoreceptors from dissociated retinal organoids are needed to increase the potency of these treatments. In this work, we have shown that partial dissociation can be used to exploit the structural organization of retinal organoids to produce highly pure populations of dissociated photoreceptor cells without the use of specialized equipment or antibody-based labels. While our initial results have been encouraging, further development is needed to ensure that this approach produces consistent results across lines derived from the many patients in need of photoreceptor cell transplants. The most obvious limitation of our partial dissociation technique is that its performance is dependent on the degree of organization and abundance of each cell type present in the organoids of interest. While we have shown that this issue can be overcome by simply preselecting organoids with obvious lamination and absence of disorganized appendages, the level of waste resulting from this strategy is not desirable. This is especially true as large numbers of differentiations need to be performed in parallel to produce autologous therapies for many patients in a clinical setting. Therefore, we and others have been investigating protocol changes aimed at producing highly organized retinal organoids. For instance, by using 96-well, U-bottom plates, Harkin et al. recently demonstrated a highly effective iPSC reaggregation strategy that enabled production of high-quality uniform embryoid bodies (EBs). By plating these EBs on an adherent 2D surface and allowing for a short period of expansion and retinal cell fate commitment, the authors were able to pick optic vesicles and generate laminated retinal organoids with a uniform shape and size from all iPSC lines evaluated (27).

Given optimal organoid structure, an additional consideration is that organoids produced from different patients or rounds of differentiation may contain differing proportions of photoreceptors or dissociate at different rates. In this case, simply dissociating organoids for the same amount of time may not be sufficient to ensure that the composition of liberated cells is consistent across patients and manufacturing runs. However, our initial results indicate that there may be another indicator that could be used to control for batch-to-batch variability in dissociation rate. In all the dissociations that we performed on well-organized retinal organoids, dissociation rate first decreased from its initial value before increasing. This suggests that dissociation kinetics could be used to monitor the process in a time-independent manner, allowing for production of consistent cell populations across patients and rounds of differentiation.

While retinal organoids are an ideal source of cells for autologous photoreceptor cell replacement, the fact that state-of-the-art differentiation strategies result in complex tissues containing multiple cell types complicates their potential clinical use. To ensure uniform, positive treatment outcomes, it is important that consistent cell populations be used for transplant and that these populations only contain cells expected to contribute to the recovery of vision. While the need for clinically compatible photoreceptor enrichment strategies is clear, most traditional approaches have significant drawbacks. FACS and MACS, while mature technologies, require the use of expensive equipment and the exposure of cells destined for transplant to antibody-conjugated labels. In this work, we demonstrate how partial dissociation can be used to exploit the structure of iPSC-derived retinal organoids to produce highly pure photoreceptor cells without using specialized equipment or reagents such as antibody tags. Although our current protocols do not always produce perfectly laminated organoids from every line of patient-derived iPSCs, protocol improvements such as presorting of laminated organoids from populations of mixed quality show promise for increasing the performance and applicability of this technique. In addition, we believe that monitoring dissociation kinetics will provide additional feedback to reduce line-to-line variability and provide additional documentation as to the integrity of the cell fractions liberated. In summary, partial dissociation constitutes a cheap, clinically translatable, and effective technique for purification of transplantable photoreceptor cells.

## Methods

### Sex as a biological variable.

To control for sex as a biological variable, iPSC lines were derived from both male and female donors. This study included 2 female donors and 3 male donors.

### Patient-derived iPSC generation and validation.

iPSCs were generated and validated as described previously (28). Briefly, fibroblasts from 4 patients (as described in Supplemental Table 1) were isolated from 3 mm dermal punch biopsies and reprogrammed using the CytoTune2 kit (Thermo Fisher Scientific, A16517), a nonintegrating Sendai viral reprogramming kit. At passage 10–12, iPSC lines were subject to karyotyping and TaqMan Human Pluripotent Stem Cell Scorecard Panel (Thermo Fisher Scientific, A15870) to confirm absence of transgene expression, genetic integrity, and potency.

### Retinal organoid differentiation.

Retinal differentiation was performed as described previously with minor modifications (29, 30). Briefly, iPSCs were cultured on rLaminin-521–coated (BioLamina, 354224) plates in E8 medium (Thermo Fisher Scientific, A1517001). IPSCs were lifted with ReLeSR (STEMCELL Technologies, 05872) and transitioned from E8 to neural induction medium — NIM-DMEM/F12 (1:1; Thermo Fisher Scientific, 11320033), 1% N2 supplement (Thermo Fisher Scientific, 17502048), 1% nonessential amino acids (Thermo Fisher Scientific, 11140050), 1% GlutaMAX (Thermo Fisher Scientific, 35050061), 2 μg/mL heparin (Sigma-Aldrich, H3149), and 0.2% Primocin (InvivoGen, ant-pm-2) — over a 4-day period. On day 6, NIM was supplemented with 1.5 nM rhBMP4 (R&D Systems, 314-BP-05/CF). On day 7, EBs were adhered to CELLstart-coated plates (Thermo Fisher Scientific, A1014201). BMP4 was gradually transitioned out of the NIM over 7 days. On day 16, the media were changed to retinal differentiation medium: RDM-DMEM/F12 3:1, 2% B27 supplement (Thermo Fisher Scientific, 17504044), 1% nonessential amino acids, 1% GlutaMAX, and 0.2% Primocin. On day 25–30, the entire EB outgrowth was mechanically lifted and transferred to ultra-low-attachment flasks in 3D-RDM (RDM plus 10% FBS, Atlas Biologicals, F-0500-A; Thermo Fisher Scientific), 100 μM taurine (Sigma-Aldrich, T0625), 1:1,000 chemically defined lipid concentrate (Thermo Fisher Scientific, 11905031), and 1 μM all-trans retinoic acid (until day 100; Sigma-Aldrich, R2625). The cells were fed 3 times per week with 3D-RDM until harvest at day 160.

### Staged partial dissociation.

For each partial dissociation experiment, organoids were settled and resuspended in 0.5 mL of dissociation solution consisting of 30 U/mL papain (Worthington; LK003176) and 120 U/mL DNase (Worthington; LS006361) in Earle’s balanced salt solution (EBSS) (Worthington; LK003188). Tubes were placed in a rotating incubator at 37°C. At time points dependent on the experiment, the organoids being partially dissociated were allowed to settle, after which the supernatant containing liberated cells was collected. These organoids were washed once with dissociation solution, after which fresh dissociation solution was added. After the last time point was collected, any remaining solid organoid material was fully dissociated via trituration (i.e., mechanical aspiration and expulsion via a 1 mL pipette). The control dissociations were incubated uninterrupted for 60 minutes, after which any remaining organoid mass was fully dissociated. After dissociating the organoids, collected cells were passed through a 40 μm cell strainer.

### Staged partial dissociation for CytoSpin.

Approximately 15 organoids were selected for staged partial dissociation along with 2 organoids, which were dissociated in 1 step as a control. Cells were collected from the partially dissociated organoids after 20, 40, and 60 minutes, and the remaining solid aggregates were fully dissociated. Cells were loaded into EZ Single Cytofunnel consumables (Epredia, A78710004) and spun onto slides using an Epredia CytoSpin 4 centrifuge (Thermo Fisher Scientific, A78300003) at 254*g* for 5 minutes. The cells were fixed in 4% paraformaldehyde (PFA) for 10 minutes and stained as described below. Photoreceptor purity (percentage of cells NRL^+^ or ARR3^+^) was assessed by a skilled reader who was masked to the experimental conditions. Four separate regions per condition were counted.

### Staged partial dissociation for qPCR.

Approximately 30 organoids were selected for staged partial dissociation. Liberated cells were collected at 20, 40, 60, and 80 minutes, after which any remaining tissue was fully dissociated. After collection, cells were pelleted and RNA was extracted using NucleoSpin columns (Macherey-Nagel, 740955). cDNA was synthesized using Superscript VILO master mix (Thermo Fisher Scientific, 11754050). qPCR was performed in triplicate using TaqMan fast advanced master mix (Thermo Fisher Scientific, 4444556) and PrimeTime assays for CRX, NRL, RCVRN, ARR3, RHO, BEST1, HNRNPL1, and 18S (IDT). Primer and probe sequences for all assays are available in Supplemental Table 7. ΔΔCT analysis was performed using the geometric mean of HNRNPL1 and 18S as a loading control, and all samples were normalized against the full dissociation group (i.e., the tissue remaining at the end of the partial dissociation).

### Staged partial dissociation for flow cytometry.

Approximately 30 organoids were selected for partial dissociation. For this experiment, organoids were placed in 2 mL of papain, and 400 μL of supernatant was collected at 30 and 60 minutes, after which remaining tissue was fully dissociated. The 3 samples were stained for the photoreceptor marker CD133 (Miltenyi Biotec, 130-111-081) and CD140b (Miltenyi Biotec, 130-131-577) and were counterstained with Hoechst (31). ICC showing that the CD133 antibody used specifically stains photoreceptors in retinal organoids is presented in Supplemental Figure 4. Samples were analyzed on an LSR-II flow cytometer (BD Biosciences). Gates were chosen to remove debris based on forward scatter intensity; autofluorescent RPE cells and debris were removed based on characteristic coemission in the V450 and AF488 channels. Putative photoreceptors were identified in the remaining events based on CD133 staining.

### Staged partial dissociation of retinal organoids for scRNA-Seq.

For each sample tested, approximately 20 organoids were selected for staged partial dissociation along with 10 organoids that were dissociated in 1 step as a control. Cells liberated via partial dissociation were collected at 20, 40, and 60 minutes, after which the remaining tissue was fully dissociated. The collected cells were then passed through a 40 μm filter and counted using a Moxi Go II counter (Orflo). All samples were pelleted and resuspended in 8 μg/mL recombinant albumin (New England Biolabs; B9200S) in dPBS^–/–^ for encapsulation with the Chromium X instrument (10X Genomics). Approximately 8,000 cells were targeted for encapsulation per sample.

### Single-cell gene expression library preparation and sequencing.

Single cells were partitioned and barcoded with the Chromium Controller instrument (10X Genomics) and Single Cell 3′ Reagent (v3.1 chemistry) kit (10X Genomics) according to the manufacturer’s specifications with no modification (protocol revision Rev E). Final libraries were quantified using the Qubit dsDNA HS Assay Kit (Thermo Fisher Scientific, Q32851) and diluted to 3 ng/μL in buffer EB (Qiagen, 19086). Library quality was confirmed using the Bioanalyzer High Sensitivity DNA Assay (Agilent, 50674626) prior to sequencing as previously described (32).

### Single-cell gene expression data integration and processing.

scRNA libraries were pooled and sequenced using the NovaSeq 6000 instrument (Illumina) generating 100 bp paired end reads. Sequencing was performed by the Genomics Division of the Iowa Institute of Human Genetics. FASTQ files were generated from base calls with the bcl2fastq software (Illumina), and reads were mapped to the prebuilt GRCh38 reference (refdata-gex-GRCh38-2020-A) with Cell Ranger v7.0.0 (10X Genomics) using the “count” function with the following parameters: --expect-cells=8000 --localcores=56. Only cells passing the default Cell Ranger call were analyzed further. All time points and control dissociations for the 4 samples studied (line 3, line 4, and the well-organized and poorly organized subpopulations of line 5) were integrated using canonical correlation analysis (CCA) in Seurat v4.0.3 (33). Only cells with between 2,000 and 6,000 unique genes (features) were included in the analysis to exclude low-quality cell fragments and doublets or multiplets with high feature counts. Only cells with < 10% of reads mapping to mtDNA-encoded genes were included. Counts data were normalized using the NormalizeData function (Seurat) with the following parameters: normalization.method = “LogNormalize,” scale.factor = 10000. In total, 2,000 variable features were identified with the FindVariableFeatures function using the vst selection method. Integration anchors were identified using the FindIntegrationAnchors function using 25 dimensions. An assay “Integrated” was generated for the 2,000 variable features using the IntegrateData function using 25 dimensions.

### Dimensionality reduction with UMAP and cell type annotation.

Thirty principal components were identified out of the integrated dataset described above using the RunPCA function (Seurat) (33). Uniform manifold approximation and projection (UMAP) was performed using the RunUMAP function using 25 principal components. Cells were then clustered using Seurat’s FindNeighbors and FindClusters functions with the following parameters: k.param = 20, reduction = ”pca”, dims = 1:25 (for FindNeighbors), and resolution = 0.5 (FindClusters) using the original Louvain algorithm as implemented by Seurat. As shown in Supplemental Figure 1, the expression patterns of genes previously shown to be markers of retinal organoid cell types were then examined in these clusters and were used to manually annotate each cluster with a cell type (34). In all calculations of photoreceptor purity, cells contained in clusters that highly express CRX were counted as fate-committed photoreceptors, and all other clusters were treated as nonphotoreceptor cells. Dot plot of cluster expression was generated using scCustomize (35).

### ICC.

Day 160 organoids were fixed with 4% PFA for 30–60 minutes at room temperature and equilibrated to 15% sucrose in PBS, followed by 30% sucrose. Organoids were cryopreserved in 50:50 solution of 30% sucrose/PBS:tissue freezing medium (Electron Microscopy Sciences, 72592) and cryosectioned (15 μm). Sections were blocked with 5% normal donkey serum, 3% BSA, and 0.1% Triton X-100 and were stained overnight with the primary antibodies listed in Supplemental Table 2. Secondary antibodies (Supplemental Table 2) were incubated for 1 hour, and cell nuclei were counterstained using DAPI (Thermo Fisher Scientific, 62248).

### Data visualization.

Plots for Figure 2 and Figure 3 were created using the Julia packages CSVFiles.jl, FileIO.jl, DataFrames.jl, StatsPlots.jl, ImageIO.jl, Images.jl, and Colors.jl (36–38).

### Data availability.

Raw and processed data from single-cell experiments are available on GEO under accession no. GSE266526. Values for all data points in graphs are reported in the Supporting Data Values file.

### Study approval.

This study was approved by the IRB of the University of Iowa (project approval no. 200202022) and adhered to the tenets set forth in the Declaration of Helsinki. Written informed consent was obtained from all individuals prior to participation.

## Author contributions

NES, LRB, AB, and BAT designed and performed experiments. NKM performed scRNA-Seq library preparation and data analysis. ATW and AB performed IHC. EMS provided patient samples for iPSC generation. RFM and ICH assisted with study design and reviewed the manuscript. BAT, RFM, and EMS provided financial support.

## Supplementary Material

Supplemental data

Supporting data values

## Version 1 06/12/2025

In-Press Preview

## Version 2 07/22/2025

Electronic publication
