# Supplementary material for: Device-free isolation of photoreceptor cells from patient iPSC-derived retinal organoids
Source: JCI Insight. 2025 Jun 12;10(14):e186338. doi: 10.1172/jci.insight.186338 (PMC12288960; doi:10.1172/jci.insight.186338)
Supplement: Supplemental data [file jciinsight-10-186338-s090.pdf]

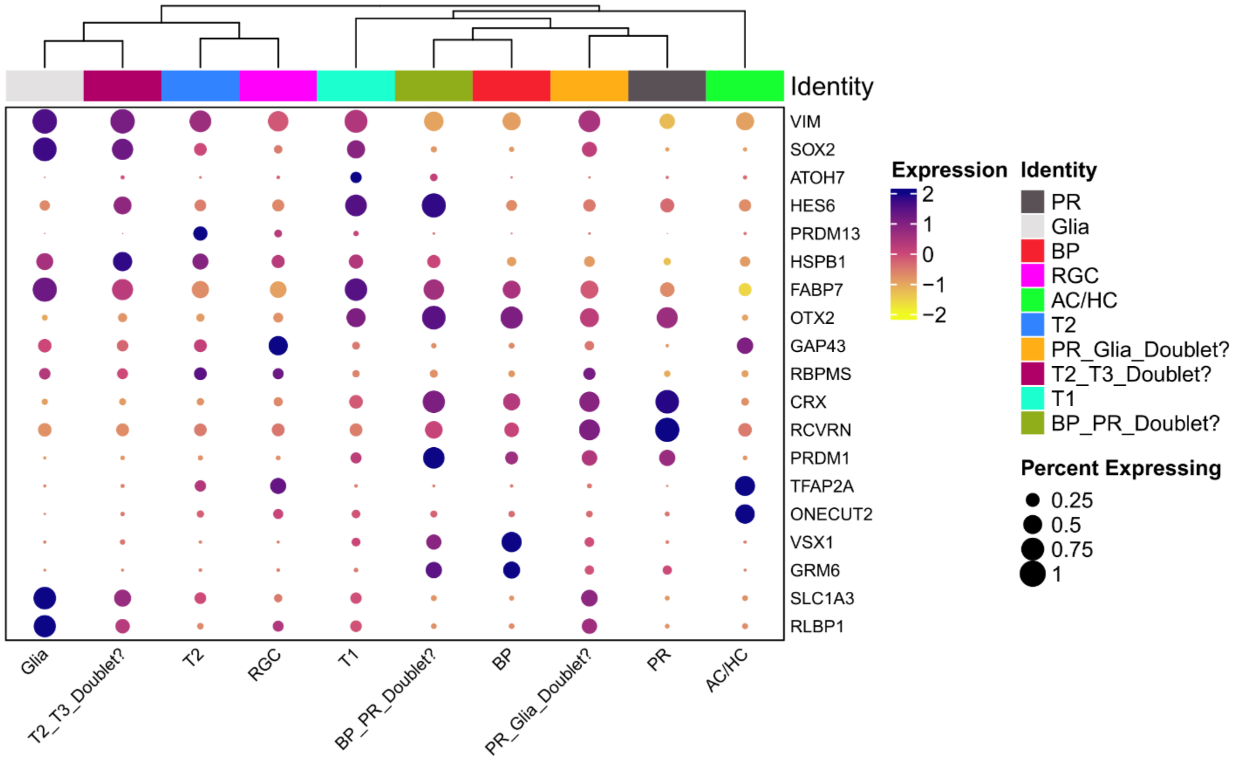

Supplemental Figure S1: Gene expression and cell type annotations for clusters identified by Seurat. Fate-committed photoreceptor clusters were identified through their expression of canonical markers such as CRX and RCVRN.

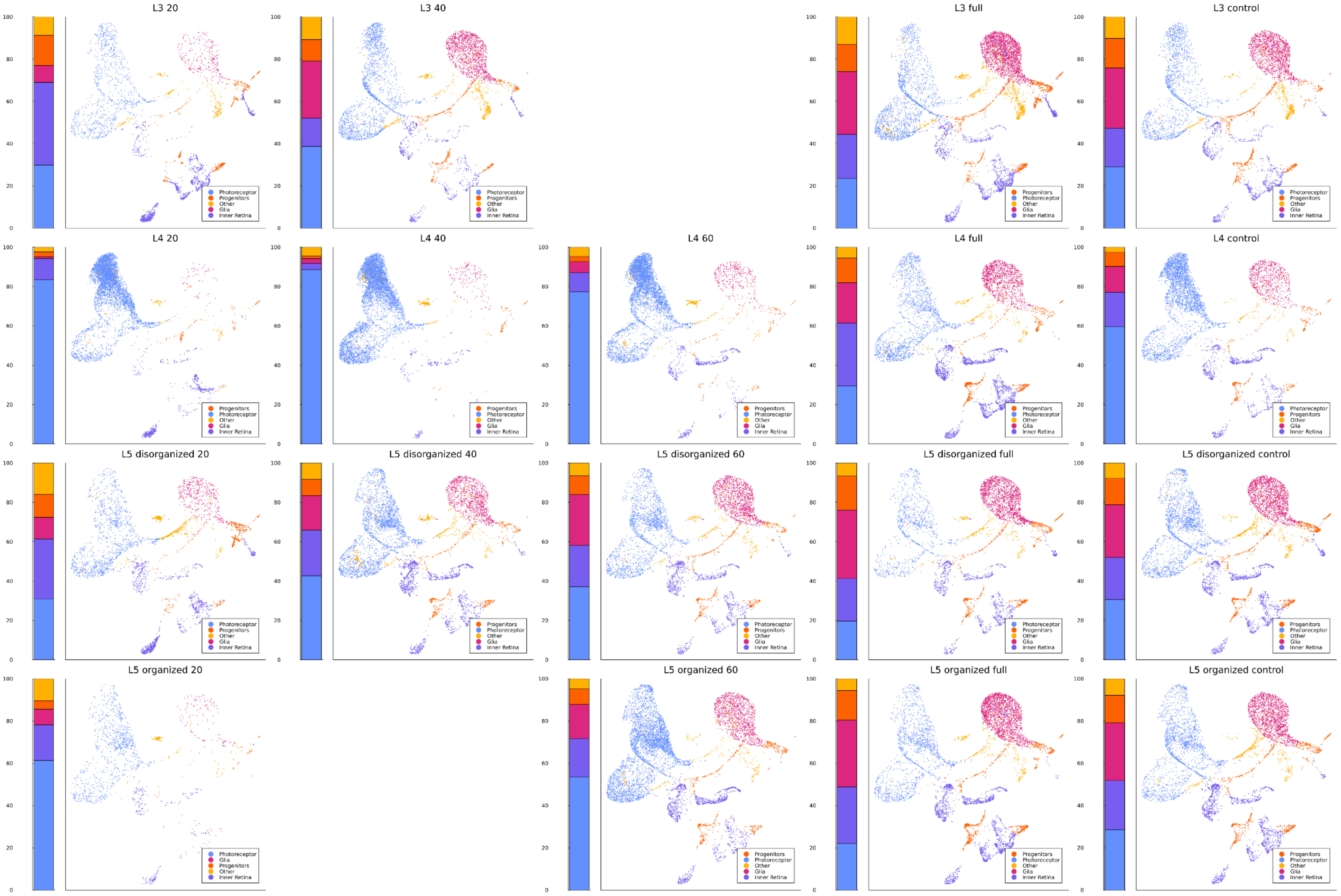

Supplemental Figure S2: UMAP plots for all scRNAseq samples.

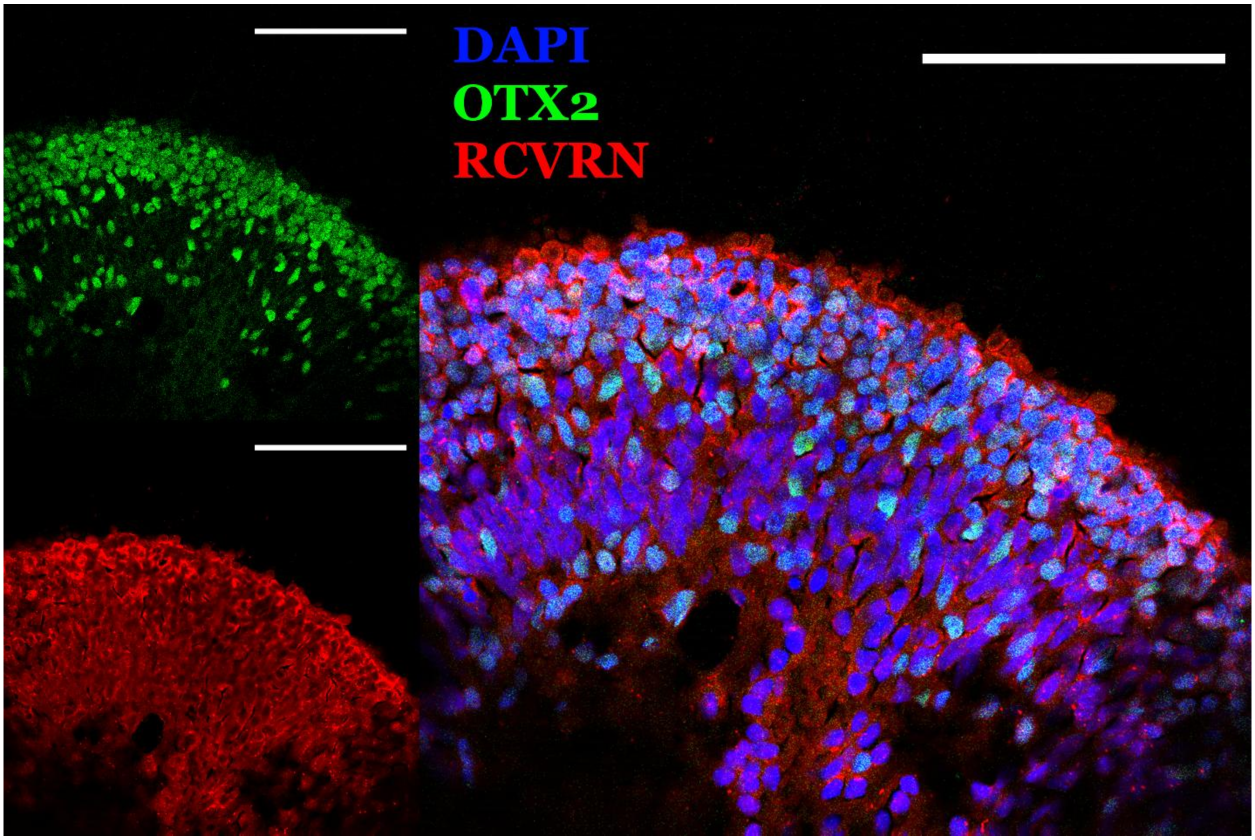

Supplemental figure S3: ICC of D160 line 5 organoids. Scale bars = 100μm.

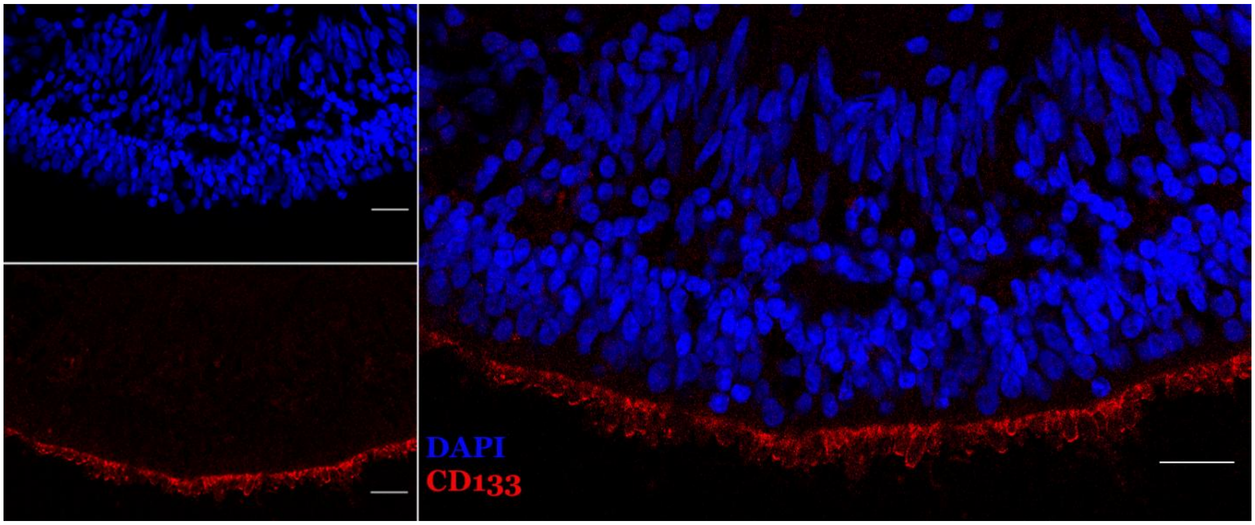

Supplemental figure S4: ICC of D260 line 4 organoids showing specific staining of photoreceptors by the CD133 antibody used in the flow cytometry experiment presented in Figure 3. Scale bars = 25µm.

Supplemental table S1, cell lines used

Line number	Identifier	Sex	Genotype
1	B342	F	Homozygous for c.119-2A>C mutation in NR2E3
2	B1771	M	Homozygous for c.1513ins353 in MAK
3	B1427	M	No disease control
4	B342cor	F	Isogenic control for line 1
5	B1737	M	Compound heterozygous for c.932G>A; p.(Arg311Gln) and c.119-2A>C mutations in NR2E3

Table S2: Antibodies used

Primary Antibodies			
Target	Host	Vendor	Catalog number
ARR3	Rabbit	LifeSpan Bio	LS-C368677
NRL	Goat	R&D Systems	AF2945
OTX2	Goat	R&D Systems	AF1979
Recoverin (RCVRN)	Rabbit	Millipore	AB5585
Secondary Antibodies			
Donkey anti-goat 488		Thermo	A11055
Donkey anti-rabbit 647		Thermo	A31573

Supplemental Table S3: Cell counts obtained by an expert reader assessing ICC of partially dissociated cell subjected to CytoSpin.

spin 1				
Sample	DAPI	NRL	ARR3	%PR
Control	147	48	31	53.7414966
Full	356	56	45	28.3707865
20 min	56	30	14	78.5714286
40 min	116	61	39	86.2068966
60 min	326	222	40	80.3680982
spin 2				
Sample	DAPI	NRL	ARR3	%PR
Control	74	30	13	58.1081081
Full	280	55	25	28.5714286
20 min	119	65	24	74.789916
40 min	165	88	34	73.9393939
60 min	71	37	18	77.4647887
spin 3				
Sample	DAPI	NRL	ARR3	%PR
Control	155	53	22	48.3870968
Full	241	30	35	26.9709544
20 min	84	59	20	94.047619
40 min	183	109	41	81.9672131
60 min	389	181	53	60.1542416
spin 4				
Sample	DAPI	NRL	ARR3	%PR
Control	92	26	17	46.7391304
Full	262	40	39	30.1526718
20 min	125	82	18	80
40 min	128	69	31	78.125
60 min	353	150	49	56.3739377

Supplemental Table S4: qPCR gene expression data. CT values represent the mean of three technical replicates

sample	target	ct	loading ct	dct	dct_fold	control dct	ddct	ddct_fold
20	NRL	29.13	18.81	10.32	7.83E-04	13.42	-3.10	8.57E+00
40	NRL	30.11	18.70	11.40	3.69E-04	13.42	-2.01	4.04E+00
60	NRL	30.81	18.71	12.10	2.28E-04	13.42	-1.32	2.49E+00
80	NRL	31.90	18.78	13.12	1.12E-04	13.42	-0.30	1.23E+00
full	NRL	32.66	19.25	13.42	9.14E-05	13.42	0.00	1.00E+00
20	CRX	27.47	18.81	8.66	2.47E-03	11.90	-3.24	9.45E+00
40	CRX	28.41	18.70	9.70	1.20E-03	11.90	-2.20	4.59E+00

60	CRX	29.43	18.71	10.72	5.95E-04	11.90	-1.19	2.28E+00
80	CRX	30.35	18.78	11.57	3.29E-04	11.90	-0.33	1.26E+00
full	CRX	31.15	19.25	11.90	2.61E-04	11.90	0.00	1.00E+00
20	BEST1	29.24	18.81	10.43	7.26E-04	8.63	1.80	2.87E-01
40	BEST1	28.32	18.70	9.62	1.27E-03	8.63	0.99	5.04E-01
60	BEST1	28.34	18.71	9.63	1.27E-03	8.63	1.00	5.01E-01
80	BEST1	27.47	18.78	8.69	2.41E-03	8.63	0.07	9.55E-01
full	BEST1	27.88	19.25	8.63	2.53E-03	8.63	0.00	1.00E+00
20	ARR3	27.89	18.81	9.08	1.84E-03	13.69	-4.61	2.44E+01
40	ARR3	29.86	18.70	11.16	4.37E-04	13.69	-2.53	5.79E+00
60	ARR3	30.57	18.71	11.85	2.70E-04	13.69	-1.84	3.58E+00
80	ARR3	31.63	18.78	12.85	1.36E-04	13.69	-0.85	1.80E+00
full	ARR3	32.94	19.25	13.69	7.55E-05	13.69	0.00	1.00E+00
20	RHO	28.77	18.81	9.96	1.00E-03	13.78	-3.82	1.41E+01
40	RHO	29.61	18.70	10.91	5.20E-04	13.78	-2.87	7.30E+00
60	RHO	30.53	18.71	11.82	2.76E-04	13.78	-1.96	3.88E+00
80	RHO	31.76	18.78	12.98	1.24E-04	13.78	-0.80	1.74E+00
full	RHO	33.02	19.25	13.78	7.12E-05	13.78	0.00	1.00E+00
20	RCVRN	25.24	18.81	6.43	1.16E-02	9.26	-2.84	7.15E+00
40	RCVRN	26.07	18.70	7.37	6.04E-03	9.26	-1.89	3.71E+00
60	RCVRN	26.68	18.71	7.97	3.99E-03	9.26	-1.29	2.45E+00
80	RCVRN	27.89	18.78	9.11	1.81E-03	9.26	-0.15	1.11E+00
full	RCVRN	28.51	19.25	9.26	1.63E-03	9.26	0.00	1.00E+00
20	HNRNPL1	29.14	18.81	10.33	7.77E-04	10.42	-0.09	1.06E+00
40	HNRNPL1	28.74	18.70	10.03	9.56E-04	10.42	-0.39	1.31E+00
60	HNRNPL1	28.67	18.71	9.96	1.01E-03	10.42	-0.46	1.38E+00
80	HNRNPL1	28.95	18.78	10.17	8.71E-04	10.42	-0.25	1.19E+00
full	HNRNPL1	29.66	19.25	10.42	7.31E-04	10.42	0.00	1.00E+00
20	18S	12.14	18.81	-6.67	1.02E+02	-6.76	0.09	9.39E-01
40	18S	12.18	18.70	-6.53	9.24E+01	-6.76	0.23	8.53E-01
60	18S	12.21	18.71	-6.50	9.04E+01	-6.76	0.26	8.35E-01
80	18S	12.19	18.78	-6.60	9.67E+01	-6.76	0.16	8.93E-01
full	18S	12.49	19.25	-6.76	1.08E+02	-6.76	0.00	1.00E+00

Supplemental Table S5: Relative cell type proportions and enrichment

line	celltype	count	prop	timepoint	control_prop	enrichment
4	Photoreceptor	4800	83.38	20	59.69037	1.40
4	Inner Retina	621	10.79	20	17.39297	0.62
4	Progenitors	143	2.48	20	7.186544	0.35
4	Other	142	2.47	20	2.561162	0.96
4	Glia	51	0.89	20	13.16896	0.07
4	Photoreceptor	4481	77.26	60	59.69037	1.29
4	Inner Retina	568	9.79	60	17.39297	0.56
4	Progenitors	158	2.72	60	7.186544	0.38
4	Glia	320	5.52	60	13.16896	0.42
4	Other	273	4.71	60	2.561162	1.84
4	Photoreceptor	5403	88.54	40	59.69037	1.48
4	Inner Retina	200	3.28	40	17.39297	0.19
4	Other	272	4.46	40	2.561162	1.74
4	Glia	147	2.41	40	13.16896	0.18
4	Progenitors	80	1.31	40	7.186544	0.18
4	Progenitors	713	12.44	full	7.186544	1.73
4	Inner Retina	1837	32.05	full	17.39297	1.84
4	Other	318	5.55	full	2.561162	2.17
4	Photoreceptor	1687	29.44	full	59.69037	0.49
4	Glia	1176	20.52	full	13.16896	1.56
3	Inner Retina	1234	39.09	20	18.25853	2.14
3	Progenitors	452	14.32	20	14.0583	1.02
3	Photoreceptor	944	29.90	20	29.07557	1.03
3	Glia	253	8.01	20	28.44265	0.28
3	Other	274	8.68	20	10.16494	0.85
3	Glia	1308	27.08	40	28.44265	0.95
3	Other	518	10.72	40	10.16494	1.06
3	Photoreceptor	1868	38.67	40	29.07557	1.33
3	Progenitors	491	10.17	40	14.0583	0.72
3	Inner Retina	645	13.35	40	18.25853	0.73
3	Glia	2308	29.76	full	28.44265	1.05
3	Other	1004	12.94	full	10.16494	1.27
3	Inner Retina	1618	20.86	full	18.25853	1.14
3	Photoreceptor	1820	23.47	full	29.07557	0.81
3	Progenitors	1006	12.97	full	14.0583	0.92
5 organized	Photoreceptor	938	61.39	20	28.57143	2.15
5 organized	Other	158	10.34	20	7.873377	1.31
5 organized	Inner Retina	256	16.75	20	23.21429	0.72
5 organized	Glia	113	7.40	20	27.38636	0.27
5 organized	Progenitors	63	4.12	20	12.95455	0.32

5 organized	Progenitors	539	7.38	60	12.95455	0.57
5 organized	Photoreceptor	3905	53.49	60	28.57143	1.87
5 organized	Inner Retina	1328	18.19	60	23.21429	0.78
5 organized	Glia	1178	16.14	60	27.38636	0.59
5 organized	Other	350	4.79	60	7.873377	0.61
5 organized	Inner Retina	1759	26.78	full	23.21429	1.15
5 organized	Photoreceptor	1448	22.05	full	28.57143	0.77
5 organized	Glia	2082	31.70	full	27.38636	1.16
5 organized	Progenitors	911	13.87	full	12.95455	1.07
5 organized	Other	368	5.60	full	7.873377	0.71
5 disorganized	Other	603	15.89	20	7.842525	2.03
5 disorganized	Progenitors	448	11.81	20	13.34167	0.88
5 disorganized	Inner Retina	1160	30.57	20	21.41853	1.43
5 disorganized	Glia	415	10.94	20	26.83956	0.41
5 disorganized	Photoreceptor	1169	30.80	20	30.55773	1.01
5 disorganized	Photoreceptor	1829	37.17	60	30.55773	1.22
5 disorganized	Progenitors	469	9.53	60	13.34167	0.71
5 disorganized	Glia	1271	25.83	60	26.83956	0.96
5 disorganized	Inner Retina	1029	20.91	60	21.41853	0.98
5 disorganized	Other	322	6.54	60	7.842525	0.83
5 disorganized	Progenitors	473	8.13	40	13.34167	0.61
5 disorganized	Glia	1026	17.64	40	26.83956	0.66
5 disorganized	Inner Retina	1353	23.26	40	21.41853	1.09
5 disorganized	Photoreceptor	2476	42.56	40	30.55773	1.39
5 disorganized	Other	489	8.41	40	7.842525	1.07
5 disorganized	Photoreceptor	912	19.62	full	30.55773	0.64
5 disorganized	Progenitors	803	17.27	full	13.34167	1.29
5 disorganized	Glia	1621	34.87	full	26.83956	1.30
5 disorganized	Inner Retina	1005	21.62	full	21.41853	1.01
5 disorganized	Other	308	6.63	full	7.842525	0.84

Supplemental Table S6: Concentrations of cell fractions obtained during scRNAseq partial dissociation experiments.

Line	Timepoint	cell concentration (cells/mL)
3	20	8.30E+06
4	20	5.41E+06
3	40	1.81E+06
4	40	1.63E+06
4	60	3.23E+06
3	60	1.30E+06
4	60_full	7.77E+06

3	60_full	2.18E+06
4	60_control	4.21E+06
3	60_control	2.86E+06
5-disorganized	20	4.16E+05
5-organized	20	5.62E+05
5-disorganized	40	9.27E+05
5-organized	40	4.69E+04
5-disorganized	60	1.70E+06
5-organized	60	2.43E+06
5-disorganized	60_full	2.71E+06
5-organized	60_full	2.84E+06
5-disorganized	60_control	4.32E+06
5-organized	60_control	2.38E+06

Supplemental Table S7: PrimeTime assay sequences

Gene	Primer 1	Primer 2	Probe
18S	CACGGACAGGATTGACAGATT	GCCAGAGTCTCGTTCGTTATC	AGTTGGTGGAGCGATTTGTCTGGT
HNRNPL	AATGGAGTTCAGGCGATGG	GCTTTGCGTATTCGATCTTCAG	TGACTCAGTTCAAAGTGCCCAGCG
CRX	CTCCAGCTCCTCCAGTTG	ACTATTCTGTCAACGCCTTGG	AAGTGGCCCCAGTGTGGATCTG
NRL	GGTTCCCGCTTTACCTCA	CACTGACCACATCCTCTCG	CTCCAGAATGGCCCTGCCCC
RCVRN	GCGTGTTTTCATCGTCTGGA	CCTTCTCCCTCTACGACGTG	ACTTCATTCTTGCTGATGGTCCCGT
RHO	TTCATTCCTCCATTCCTTCCTG	CGAGGATTCTTGCTTTCTGGA	ACCTACTGTGTGCCCCATTCTGTG
ARR3	ACTATGTCCTCAGAGCTAGCG	CCTACAAAGTCAGAGTCAACCTG	AGACCTGACAGCCAGCGATGTTG
BEST1	CCAATGATGCCAGCGTGA	CCCTACACAGCTGCTTCC	CAGCCTGAACAAAGAGGAGATGGAGT
